# The Molecular Breeding of Different Ecotype *Japonica* Varieties Resistant to Rice Blast with High Genome Collinearity

**DOI:** 10.3390/plants14121836

**Published:** 2025-06-15

**Authors:** Shengyuan Zeng, Cancan Du, Yihao Yang, Qingfeng Hu, Chuang Li, Fang Feng, Min Guo, Dedao Jing, Tianzi Lin, Hongbing Gong, Changjie Yan

**Affiliations:** 1 Agricultural College, Yangzhou University, Yangzhou 225009, China; 13914590415@126.com (S.Z.); yihao.yang@yzu.edu.cn (Y.Y.); guomin@yzu.edu.cn (M.G.); 2 Zhenjiang Academic of Agricultural Sciences, Zhenjiang 212400, China; 20162804@jaas.ac.cn (C.D.); huqf2015@outlook.com (Q.H.); 13775386949@yeah.net (C.L.); jingdedao@163.com (D.J.); zjsltz1998@sohu.com (T.L.); 3 Wuhan Greenfafa Institute of Novel Genechip R&D Co., Ltd., Wuhan 430070, China; fengfang@greenfafa.com

**Keywords:** *Japonica* rice, blast resistance, yield, quality, *Pigm*, *Hd1*, variety

## Abstract

The Yangtze River Delta (YRD) is one of the most important *japonica* rice planting areas in China. Balancing the resistance, yield, and quality has always been a core issue in rice breeding due to the negative correlation among these three factors, while the broad-spectrum blast resistance gene *Piz* is closely linked with *Hd1*, the major gene regulating days to heading (DTH), and a precise combination of their beneficial alleles plays a key role in synchronously improving blast resistance and the regional adaptability of *japonica* rice in YRD. In this study, using the backcross progeny population derived from backbone parent ZD9471 and W1063, two alleles of *Hd1* were identified. Then, through molecular marker-assisted selection combined with Green Super Rice 40K (GSR40K) chip-based screening, six introgression lines (ILs) with two different alleles combinations of *Hd1* and *Pigm* were obtained. An evaluation of the blast resistance, yield, and quality traits showed that compared with the recipient parent, the panicle blast resistance of ILs was significantly enhanced; the grain number per panicle increased consistently with the delaying of the growth period, leading to higher yield in the ILs; the grain quality were synchronously improved. Two representative lines with similar genetic backgrounds but a significantly different regional adaptability, exhibiting a good blast resistance, high yield, and prominent quality were approved and demonstrated promising application prospects.

## 1. Introduction

The planting area of *japonica* rice in the YRD is approximately three million hectares annually, with an annual production of about 18 million tones, contributing ~25% and ~30% of China’s total *japonica* rice planting area and production, respectively, making it the most important *japonica* rice planting region in China, defined as “southern *japonica* rice” [1,2]. In recent years, with changes in farming systems and climatic conditions, rice blast has become the primary disease in this region [3,4]. For example, in 2014, the area affected by rice blast in Jiangsu Province alone reached 1.287 million hectares, and the affected area demonstrated a consistent annual increase, severely threatening rice production safety [5]. The resistance genes in current varieties in YRD are insufficient to control the occurrence of rice blast, necessitating the introduction of new resistance genes [6,7]. Utilizing broad-spectrum and durable resistance genes to breed resistant varieties is a key strategy in blast resistance breeding [8]. *Pigm*, which is the allele of *Pi2*, *Pi9*, *Pizt*, *Pi26*, *Pi40*, and *Pi50*, harbored at the *Piz* locus [9,10], has been proven high and broad-spectrum resistant to rice blast. Moreover, *Pigm* is almost absent in *japonica* rice, indicating that it holds great value in blast resistance breeding for *japonica* rice [11,12,13,14,15].

The DTH is crucial for determining the geographic adaptability (ecotype) and yield potential of rice. Over the past two decades, a series of photoperiod sensitivity/DTH genes (or QTLs) have been cloned, and the molecular regulatory networks of photoperiod signals centered on *Hd1* and *EHd1* have been preliminarily elucidated [16,17]. Studies have demonstrated that the allelic variations and interactions of *Hd4*/*Ghd7*, *Hd5*/*Ghd8*/*DTH8*, and *Hd2*/*Dth7.1*/*OsPRR37* with *Hd1* largely determine the ecotypes of rice varieties in southern China [18,19]. *Hd1* is a key gene regulating photoperiod sensitivity in rice, and there are at least 19 *Hd1* haplotypes in the main cultivated rice varieties in China, with H8 and H13 being the two main haplotypes applied in *japonica* rice varieties in China. Using preponderant *Hd1* alleles can raise the grain number per panicle, thus increasing the yield of southern *japonica* rice [20]. However, due to the strong photoperiod sensitivity of *japonica* rice varieties and the varying requirements for rice quality by people in different areas of the YRD region [21,22,23], the regional adaptability of southern *japonica* rice varieties has been largely restricted. Taking Jiangsu Province, which has the largest rice planting area (~2.2 million hectares) in the YRD as an example, there are generally four ecotypes of *japonica* rice varieties that span the latitude 30°45′ N to 35°08′ N: medium-maturity middle *japonica* (MMMj), with the growth period of 145–150 days, mainly planted in northern Jiangsu, late-maturity middle *japonica* (LMMj, 151–155 days, mainly planted in central Jiangsu), early-maturity late *japonica* (EMLj, 156–160 days, mainly planted in southern Jiangsu), and medium-maturity late *japonica* (MMLj, ≥161 days, mainly planted in southern Jiangsu and Shanghai). In northern Jiangsu, people generally prefer varieties with a medium-low amylose content (about 13% to 18%, carrying the *Wx^b^* genotype), while in central and southern Jiangsu, people prefer varieties called ‘semi-glutinous or soft rice’ with a low amylose content (8% to 13%, carrying the *Wx^mq^* genotype). The regional adaptability of each type of variety is relatively limited.

Because the *Hd1* and *Pigm* are both located on the short arm of chromosome 6, only about 1.1 Mb apart, it is hard to break their linkage drag by conventional breeding. Thus, enhancing the blast resistance of YRD *japonica* rice varieties while widening their geographic adaptation range is a large challenge in rice breeding. Identifying more favorable *Hd1* alleles and using molecular breeding to break the *Pigm-Hd1* linkage is highly valuable for simultaneously improving the blast resistance and adaptation of southern *japonica* rice [24,25]. In this study, employing the backcross progeny population derived from ZD9471 and W1063, an *indica-japonica* intermediate line carrying the *Pigm Hd1* was identified as the gene regulating DTH in the BC_4_F_2_ population through bulked segregant analysis (BSA) based on the GSR40K chip and gene sequencing. ILs with two *Hd1* alleles recombined with *Pigm* were obtained. The blast resistance, yield, yield components, and quality of the ILs were investigated to explore their breeding value, and new blast-resistant *japonica* rice germplasm resources suitable for different regions in YRD were developed.

## 2. Results

### 2.1. Hd1 Leads to Significant Differences in DTH

**Figure 1:**
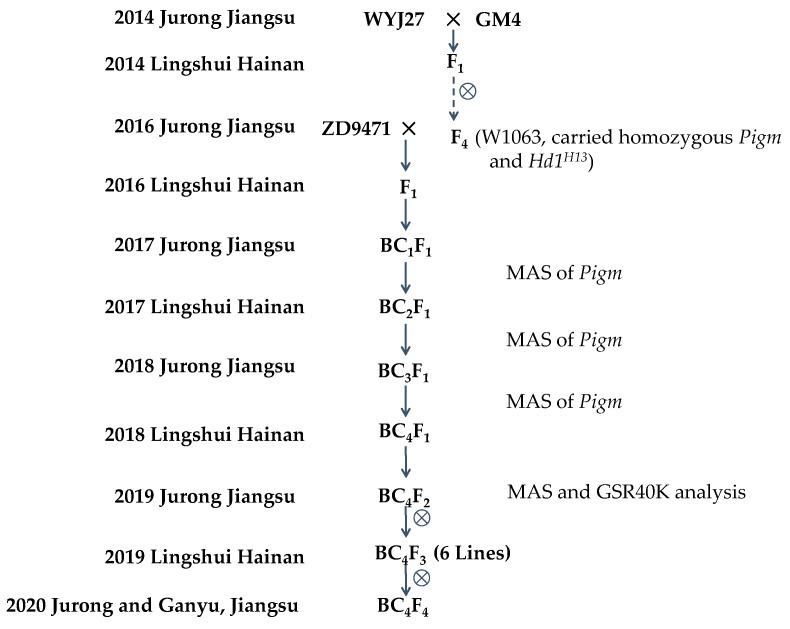
Generations of different ecotype *japonica* varieties resistant to rice blast in this study.

**Figure 2:**
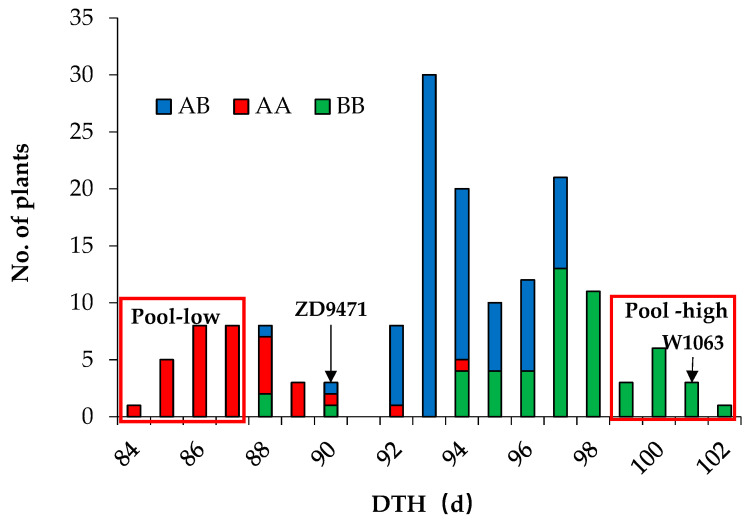
The distribution of the DTH of the BC_4_F_2_ individuals. AA (the red color) represents the homozygous genotype of ZD9471 (*pigm/pigm*) genotyped by the Pigm-4 marker, BB (the green color) represents the homozygous genotype (*Pigm/Pigm*) of W1063, and AB (the blue color) represents the heterozygous genotype (*Pigm/pigm*), respectively. The individual plants in the red box indicates that they have been selected for constructing the mixed pools.

The breeding process employed in this study is shown in Figure 1. In the 2019 growing season in Jurong, the DTH of 165 individuals of the BC_4_F_2_ population were investigated, and a significant segregation in DTH was observed. Set the threshold at 91 days, and the ratio of early-heading individuals to late-heading individuals was 38:127, fitting a 1:3 segregation ratio (χ^2^_1:3_ = 0.74 < 3.84), indicating the presence of a major gene controlling DTH which is tightly linked to the Pigm-4 marker (Figure 2).

**Figure 3:**
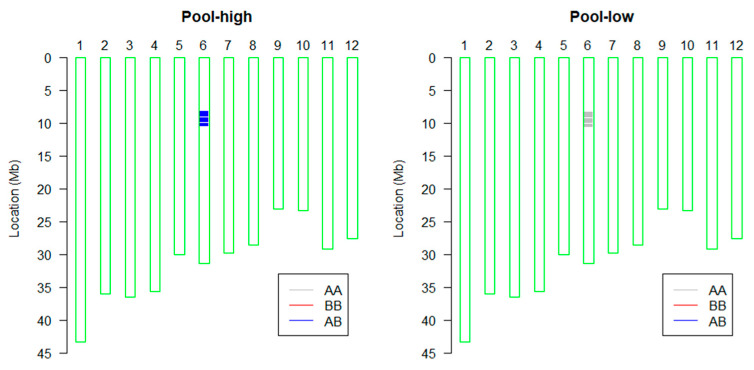
The BSA mapping based on GSR40K. “Pool-high” represents the late-heading mixed pool, “pool-low” represents the early-heading mixed pool. The green box represents the 12 chromosomes of rice. The AA (gray color) of the pool-low represents the homozygous genotype of ZD9471, the AB (blue color) of the pool-high represents the heterozygous genotype, and BB represents the homozygous genotype of W1063, while the absence of color within the green box indicates no polymorphism between the pool-high and pool-low pools.

According to the principle of BSA based on the GSR40K chip, fifteen extreme early-heading and fifteen extreme late-heading individuals were selected and mixed pooled to form low-value (pool-low) and high-value pools (pool-high), respectively (Figure 2). The two parents, ZD94171 and W1063, and the two mixed pools were used for BSA. Results show that the DTH gene was mapped on chromosome 6, between the SNPs F0607908805GA and R0611081997GA, corresponding to a physical distance of 7,908,805 bp to 11,081,997 bp (about 3.17 Mb, Figure 3). Gene annotation analysis (https://rice.uga.edu/, accessed on 1 June 2025) revealed that two reported genes *Hd1* (LOC_*Os06g16370*) and *SDG711* (LOC_*Os06g16390*) located in this region were regulating the DTH [26,27]. Therefore, these two genes were prioritized as candidate genes.

**Figure 4:**
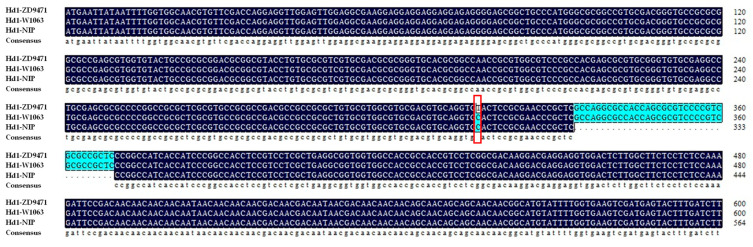
The sequence differences of *Hd1* between the two parents compared with that of Nipponbare (NIP).

Further sequencing of the recipient parent ZD9471 and the donor W1063 showed a T to C variation at 316 bp downstream of the start codon between them (Figure 4), corresponding to the H8 and H13 haplotypes of *Hd1*, respectively, as described by Leng et al., 2020 [20], while there was no sequence difference found in the mRNA of *SDG711*. In summary, we concluded that the differentiation of *Hd1* genotypes lead to the segregation of DTH in this population, and a functional marker Hd1-SNP of *Hd1* was developed.

### 2.2. The Obtaining of Introgression Lines

**Figure 5:**
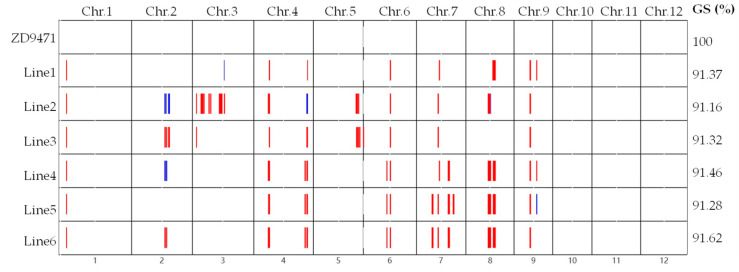
The genetic background of the six lines and receptor screened by the GSR40K chip. The red color represents a homozygous genotype different from ZD9471, while the blue color represents a heterozygous genotype different from ZD9471.

Detecting the genotypes of *Pigm* and *Hd1* by corresponding markers, a total of 41 individuals homozygous for *Pigm* were selected from 165 individuals, including three individuals with the *Pigm*/*Hd1^H8^* homozygous genotype (named as Line1–Line3) and 38 individuals with the *Pigm*/*Hd1^H13^* homozygous genotype. All of the individuals with the *Pigm*/*Hd^H8^* homozygous genotype (Line1–Line3) and the three with the highest genetic similarity to the recipient parent individuals with the *Pigm*/*Hd1^H13^* genotype (named as Line4–Line6) were selected as the target materials for further research, and the corresponding offspring (Six BC_4_F_3_ lines) were planted in Hainan. Genetic background analysis by GSR40K chip showed that the six lines exhibited a high genome collinearity (98.80–99.89%) and genetic similarity (GS) to the recipient parent of over 91.16% (Figure 5), and no other major functional genes with differences except for *Pigm* and *Hd1* were detected (Table S1), facilitating resistance, yield, and quality testing.

### 2.3. Pigm Significantly Enhances Panicle Blast Resistance

**Figure 6:**
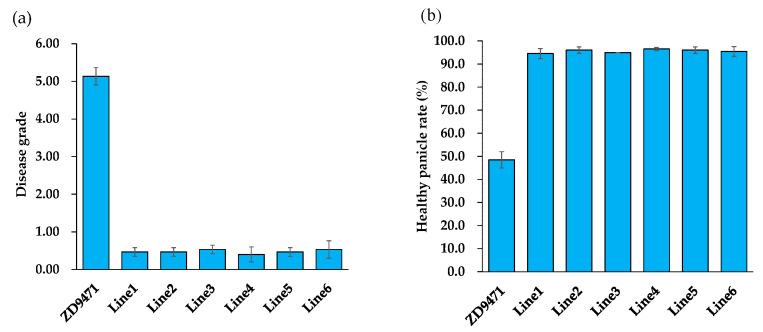
The evaluation of the panicle blast resistance of the target BC_4_F_4_ lines under artificial inoculation and blast nurseries. (**a**) The disease grade of panicle blast. (**b**) The healthy panicle rate (HPP).

The results of the artificial inoculation of panicle blast of the recipient parent and six ILs showed that the disease grade of the recipient parent was 5.13, indicating a moderate susceptibility (MS) to rice blast. In contrast, the disease grade of the ILs ranged from 0.40 to 0.53, showing resistance (R) to rice blast (Figure 6a). The healthy panicle rate (HPP) of the recipient parent in the natural nursery in Ganyu was 48.5%, while the HPP of the ILs ranged from 94.5% to 96.5% (Figure 6b), demonstrating that *Pigm* can significantly enhance the resistance of the recipient to panicle blast.

### 2.4. Agronomic and Quality Traits of Introgression Lines

Under short-day conditions in the winter of 2019 in Lingshui Hainan, the grain number per panicle (GNP) and yield per plant (YPP) significantly increased compared to the recipient parent, while other major agronomic traits, such as the DTH, plant height (PH), panicles number per plant (PN), panicle length (PL), seed setting rate (SSR), and 1000-grain weight (TGW) showed no significant differences between the six BC_4_F_3_ ILs and the recipient parent (Table 1). This is consistent with the results reported by Deng et al., 2017, that *Pigm* can increase the GNP in the recipient parent [9].

Under long-day conditions in Jurong, the DTH of the BC_4_F_4_ lines carrying the *Pigm*/*Hd1^H8^* allele were similar to the control, with significant increases in the GNP and YPP, while other agronomic traits showed no significant differences compared to the recipient parent. In contrast, the DTH of the lines carrying the *Pigm*/*Hd1^H13^* allele were significantly delayed compared to the lines carrying the *Pigm*/*Hd1^H8^* allele. The PH, PL, GNP, and YPP increased consistently with the delayed rice, as the DTH prolongs the photosynthesis period, boosting biological yield. The main agronomic traits among the ILs carrying the same *Pigm*/*Hd1* genotype combination showed little variation (Table 2), indicating that there are no other major genes that significantly affect the main agronomic traits of the ILs.

Under long-day conditions in Jurong, the quality characteristics of ILs differed significantly from the recurrent parent. Among the three ILs with the *Pigm*/*Hd1^H8^* allele, Line2 outperformed the recurrent parent, while Line1 and Line3 were similar to it, and all three ILs had a significantly lower (worsened) HRR than the control. The appearance qualities (CGP and CD) of three ILs with the *Pigm*/*Hd1^H8^* allele were better than the recurrent parent, but their rice physicochemical (AC and PC) and eating qualities were similar to it. The three ILs with the *Pigm*/*Hd1^H13^* allele showed a varying BRR, with Line4 and Line5 significantly lower and Line6 comparable to the recurrent parent. However, all three ILs had a worsened HRR, and their CGP, CD, AC and PC content were significantly lower (improved), and their eating quality was significantly better. Thus, the three ILs with the *Pigm*/*Hd1^H13^* allele showed marked improvements in appearance, physicochemical, and eating qualities (Table 3). It should be noted that *Hd1* itself usually has no direct positive effect on rice quality; however, the most suitable ecological type in south Jiangsu where this study was conducted was EMLj rice, which led to the fact that lines carrying the *Pigm*/*Hd1^H13^* allele exhibited better quality.

### 2.5. Two Representative Lines with High Yield, Good Quality, and Rice Blast Resistance Were Officially Approved

**Figure 7:**
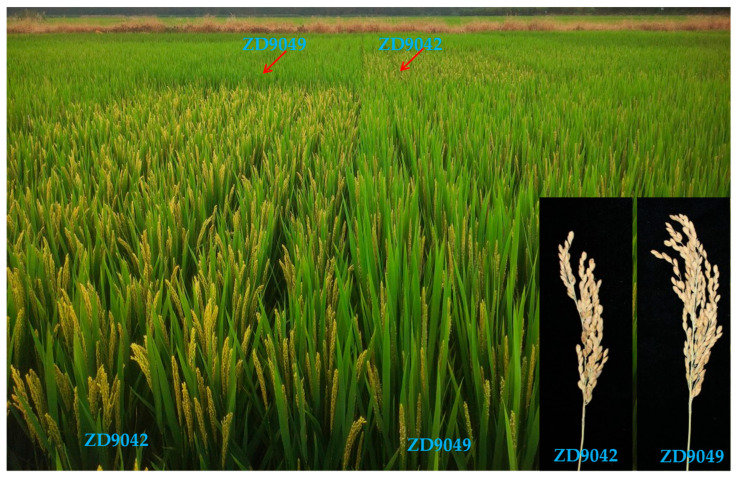
The DTH and panicle morphology of the two representative lines.

Based on the genotyping, yield, and quality analysis of the ILs, two lines with high genome collinearity but significantly different heading dates in Jiangsu (natural long days), were selected in 2021 to participate in the regional trials for MMMj (Line3, regional test name: Zhendao 9042) in northern Jiangsu and EMLj (Line6, regional test name: Zhendao 9049) in southern Jiangsu and along the Yangtze River in Jiangsu, respectively. The multi-location trial results showed that compared to ZD9471, both Zhendao 9042 and Zhendao 9049 achieved a moderate resistance level (MR) or higher level (resistance, R) to rice blast. In terms of yield traits, the two lines showed significant differences in their total growth period, GNP, and SSR, but no significant differences in other major agronomic traits. The comprehensive quality of the two lines was outstanding, reaching the national standards at Grade 2 and Grade 1, respectively (Table 4, Figure 7).

After two years of regional trials (2021–2022) and one-year concurrent production trial in 2022, Zhendao 9049 was approved in 2023 and registered as Zhendao 37 (Approval No. Sushendao 20230066), and is suitable for cultivation in southern Jiangsu and the areas along the Yangtze River in Jiangsu Province. Zhendao 9042 underwent two years of regional trials (2021–2022) and a production trial in 2023, and was approved in 2024, named Zhendao 39 (Approval No. Sushendao 20240034), and is suitable for cultivation in northern Jiangsu.

## 3. Discussion

Breeding high-quality, high-yield, multi-resistance, and broad geographic adaptive varieties is a perpetual goal in rice breeding. Numerous studies have demonstrated that *Pigm* is a broad-spectrum rice blast resistance gene that can significantly enhance blast resistance in different genetic backgrounds [9,10,11,12,13,14,15,28,29]. However, several studies have indicated that *Pigm* may also affect the agronomic traits of recipient varieties, such as reducing the 1000-grain weight while increasing grain number [9,30,31]. *Hd1* plays a crucial role in determining the rice yield potential and ecotype. Under long-day conditions, the enhanced expression of *Hd1* delaying heading, increase plant height, and the grain number per panicle, while weakened genotypes can enable rice cultivation in temperate or even colder regions [20]. However, these two key genes that determine blast resistance and growth period, are closely linked. Breaking the linkage between *Hd1* and *Pigm* and combining their superior alleles by molecular design breeding theoretically can promote the selection of materials that balance resistance, yield, and regional adaptation. In this study, two different *Hd1* alleles were rapidly uncovered by BSA mapping based on GSR40K chip and gene sequencing, and the recombination of *Hd1* with *Pigm* in the recipient parent ZD9471 were achieved through foreground selection and background screening, resulting in a series of introgression lines. Compared to the recipient parent, the panicle blast resistance of all introgression lines was significantly improved. The evaluation of the agronomic traits, yield, and quality of the introgression lines showed that the single-gene introgression lines of *Pigm* (or *Pigm/Hd1^H^^8^*) significantly increased the grain number per panicle and yield per plant, and improved the rice appearance quality, but had no significant negative impact on other agronomic traits (including 1000-grain weight) of the recipient parent. The results further demonstrate the high application value of the *Pigm* gene in *japonica* rice breeding. It can significantly improve *japonica* rice blast resistance without a yield and quality penalty if the appropriate receptor was used. The double-gene introgression lines *Pigm/Hd1^H13^* with a strong *Hd1* function showed a high genetic similarity to the single-gene introgression lines but significantly delayed the DTH, and increased plant height, grain number, and yield per plant, with a deteriorated processing quality but better appearance, physicochemical, and eating quality, achieving a simultaneous improvement in blast resistance and regional adaptability.

Overcoming the linkage drag is a concern for breeders in the process of creating improved lines, often requiring multiple backcrosses or screening large populations, which consumes significant time and effort. Moreover, several key genes controlling heading date (yield) (e.g., *Hd1*, *Hd3a*, and *Hd17*) [20,32,33], quality (*Wx* and *ALK*) [34,35], fertility (*S5*) [36], and broad-spectrum blast resistance (*Piz* locus) [9,10] are located within the dense region on the short arm of chromosome 6, where they are closely linked, making it difficult to overcome linkage drag and breed varieties that balance high quality, high yield, and multi-resistance by traditional methods. Additionally, the differentiation of the *S5* gene leads to reproductive isolation between *indica* and *japonica* subspecies, further hindering the aggregation of favorable alleles between subspecies, employing the high-density but less expensive chips can enable a precise screening of the improved lines at a lower cost in modern crop breeding. In this study, we used an *indica*-*japonica* intermediate material carrying the *Pigm* gene as a donor and backcrossed it with the elite parent ZD9471 by MAS and GSR40K chip. The preponderant recombinant individual of *Pigm* and *Hd1* were selected from the 165 BC_4_F_2_ population effectively, with improved resistance, yield, and quality compared to the recipient parent. Through multi-location testing, two different ecotype *japonica* varieties, Zhendao 37 (ZD9049, EMLj) and Zhendao 39 (ZD9042, MMLj) obtained variety certification. Zhendao 37 and Zhendao 39 exhibit high genome collinearity (identity > 98%) as determined by GSR40K chip, and only one pair of the simple sequence repeats (SSR) marker (RM547) had a difference based on 48 SSR markers followed by the protocol NY/T 1433-2014 [37], but they display a 10-day significant difference in DTH due to variations in *Hd1*. The combined planting areas of Zhendao 37 and Zhendao 39 can cover the entire middle and lower reaches of the Yangtze River region, therefore effectively overcoming the narrow regional adaptability of southern *japonica* rice varieties. This study provides a molecular design strategy and available tool (GSR40K chip) for *japonica* rice improvement. Particularly, the improved cultivars can be directly applied to rice production, providing a solid guarantee for food security.

## 4. Materials and Methods

### 4.1. Plant Materials and the Breeding Process

The rice materials and breeding process employed in this study are shown in Figure 1. The recipient parent ZD9471 is a high-yield LMMj rice variety with a good appearance quality while having a moderate susceptibility (MS) to panicle blast in field production, which was developed by Zhenjiang Academy of Agricultural Sciences. The original donor of *Pigm* is Gumei 4 (GM4), an *indica* variety that has been used for over 40 years that is durable and broad-spectrum resistant to rice blast, but its agronomic traits, especially the grain quality traits, are poor. Wuyunjing 27 (WYJ27) is a high-yield LMMj (with ~92 DTH in Jiangsu) rice variety widely planted in Jiangsu Province in 2013–2017.

In the 2014 growing season in Jiangsu, WYJ27 was crossed with GM4, and after four generations of selfing, combined with molecular marker detection, the late-maturity *japonica-indica* intermediate material W1063 (with ~101 DTH in Jiangsu) carrying homozygous *Pigm* was obtained. In the 2016 growing season, ZD9471 was used as the female parent and crossed with W1063. After four generations of backcrossing and selfing, the BC_4_F_2_ population was obtained in the 2019 growing season.

### 4.2. Identification of the DTH Gene

A total of 165 individuals from the BC_4_F_2_ population were planted, adopting conventional field management practices. The DTH of all individuals were investigated, and based on the differences in DTH, 15 early (84–87 d) and 15 late (99–102 d) extreme individuals were selected and mixed to two pools respectively (Figure 2). The GSR40K chip (Wuhan Greenfafa Institute of Novel Genechip R&D Co., Ltd., Wuhan, China) was used for the BSA analysis of the parents and the two pools.

### 4.3. Sequence Analysis

Based on the mapping results, the *Hd1* genomic DNA of ZD9471 and W1063 were amplified by segmental PCR. For the *SDG711* gene, the total RNA was extracted from the leaf at 10 days after sowing using an RNA extraction kit 298 (Tiangen, Beijing, China). First-strand cDNA was synthesized using a reverse transcription kit-299 (Vazyme, Nanjing, China), then the cDNA of ZD9471 and W1063 were amplified by PCR according to Lu et al., 2023 [38].

The PCR products were gel-purified and sent to Nanjing Qingke Biotechnology Co., Ltd. (Nanjing, China) for sequencing. The sequencing chromatograms were analyzed using Chromas 2.3.1, and the sequences were assembled and aligned using DNAMan 6.0.3.99.

### 4.4. Foreground Selection

The molecular markers for *Pigm* screening are Pigm-2 and Pigm-4, which were developed by our research group in previous studies [39]. Pigm-4 is a highly specific molecular marker located in the promoter region of the *R12* element within the *Pigm*, and Pigm-2 is located ~21.2kb upstream of the *Pigm*. The combination of these two markers ensures the integrity of the introduced donor genome fragment (Table 5). DNA extraction and molecular marker detection methods were the same as previously described [39]. Based on the alignment results of the *Hd1* gene sequence, PARMS (penta-primer amplification refractory mutation system) marker based on SNP_316_ T-C was developed and commissioned to Wuhan Gentiens Biotechnology Co., Ltd. (Wuhan, China) for detection (Table 5).

Using the above markers, 165 individuals from the BC_4_F_2_ population were genotyped, and individuals homozygous for both the *Pigm* and *Hd1* were selected for further analysis.

### 4.5. Background Screening and Analysis

The GSR40K chip was used to scan the genetic background of the target introgression lines homozygous for both genes in the BC_4_F_2_ population. The self-developed R language package RiceChipV4 was used for a genetic background analysis of the introgression lines. Genetic similarity = [1 − number of different loci/total number of loci (height)] × 100%.

### 4.6. The Evaluation of Blast Resistance

Since panicle blast is the most serious symptom of rice blast disease, it directly leads to the reduction of rice yield. The resistance to panicle blast was evaluated using both artificial inoculation and natural field nursey methods in this study.

#### 4.6.1. Artificial Inoculation

The experiment was conducted at the Base of Zhenjiang Academy of Agricultural Sciences. The test materials were sown on 20 May, and seedlings were transplanted 30 days later. Single seedlings were transplanted with five rows per plot, 12 plants per row, and a spacing of 13.3 cm × 25.0 cm. Two replicates were used, and no fungicides were used in the experimental field, and other conventional water and fertilizer management practices were followed.

According to DB32/T1123-2007 [39], the panicle blast resistance identification bacterial strains were provided by the Institute of Plant Protection, Jiangsu Academy of Agricultural Sciences, which isolated from Jiangsu Province in the previous year. Mixed strains containing seven representative *M. oryzae* isolates (ZA-ZG) were used for inoculation at the booting stage. One booting panicle was selected for each plant based on the principle that the distance between the pulvini of the flag leaf and penultimate leaf is 3–5 cm and was hence injected with 1mL conidial suspension at a concentration of 5 × 10^4^ conidia/mL [30,40], five plants per line. Inoculation was performed on 15 and 20 August, respectively, based on the differences in the growth stages of the test materials. After inoculation, the disease was assessed at the wax maturity stage (~30 days after heading) according to the ‘Technical specification for identification and evaluation of blast resistance in rice variety regional test (NY/T 2646-2014)’ [41]. The average disease grade of five inoculated panicles was used as the final disease grade for each line.

#### 4.6.2. Natural Field Nursey

In the 2020 growing season, the introgression lines were planted in the blast nurseries of Ganyu (34°93′ N, 119°17′ E), which possess suitable field conditions for blast disease development. Early-maturity lines were sown on 25 May, and late-maturity lines were sown on 25 April and raised in greenhouses. To ensure contact between each test material and the inducing varieties, a row of susceptible inducing varieties (Co39) was planted every two test plots. Field planting and management are same as the artificial inoculation described previously. The panicle blast incidence of each test material was investigated 30 days after heading. The investigation and evaluation methods followed Wu et al. [12,13]: the healthy panicle rate (HPP) = (number of panicles not infected by blast/total number of panicles in the plot) × 100%.

### 4.7. Measurement of Yield and Main Agronomic Traits

The agronomic traits of the introgression lines and the recipient parent were investigated adopting a trial design by conventional water and fertilizer management, which was performed separately from the one used for the evaluation of disease resistance. The DTH of each plant was recorded when the main panicle emerged at 1 cm, and the number of days from sowing to heading was recorded as DTH. The DTH of the population was recorded when 10–30% of the plants in the population had headed. After heading, plant height (PH) and panicles number (PN) were recorded for each test plot. At maturity, 10 plants of each replicate were sampled to investigate panicle length (PL), grain number per panicle (GNP), seed setting rate (SSR), 1000-grain weight (TGW), and yield per plant (YPP).

The harvested seeds were further used for quality measuring.

### 4.8. Measurement of Main Quality Traits

#### 4.8.1. Processing Quality

A total of 450 g of clean rice was randomly weighed from each harvested sample, and the rice husker FC2R (OTAKE, Japan) was first used to grind it into brown rice. A VP-32 (Yamamoto, Japan) type rice milling machine was used to grind the brown rice into milled rice, sift it, weigh it to calculate the milled rice rate, and keep the sample for use.

#### 4.8.2. Appearance Quality

The appearance quality of the test rice varieties, including chalky grain rate (CGR) and chalkiness degree (CD), were analyzed using the SC-E rice appearance quality analyzer (Wanshen, Hangzhou, China).

#### 4.8.3. Physicochemical Properties

The amylose content (AC) of rice was measured according to the Ministry of Agriculture standard ‘Determination of amylose content in rice– spectrophotometry method (NY/T 2639–2014)’ [42]. The protein content (PC) was measured according to the method of Yang et al., 2019 [43]. The measurements were repeated twice for each sample.

#### 4.8.4. Rice Taste Value

The taste quality of cooked rice was measured using the STA1B rice taste analyzer (Satake, Japan). According to the instructions, the appearance score (AS, total score of 10), taste score (TS, total score of 10), and comprehensive taste value (CTV, total score of 100) were read using the Chinese *japonica* rice calibration curve. The measurements were repeated three times, and the average value was taken.

### 4.9. Data Analysis

Agronomic and quality traits data were statistically analyzed by Microsoft Excel 2019 and SPSS 19.0 software. Differences were tested for significance using Dunnett’s T3 test and Tukey’s multiple range test at a level of *p* < 0.05.

## 5. Conclusions

Using the *Pigm* backcross progeny of ZD9471 as the population, this study identified two major *Hd1* genotypes (haplotypes) linked to *Pigm* via high-density chips and gene sequencing. Through MAS and background screening, ILs with *Pigm* and two different *Hd1* genotypes were obtained. These lines showed significantly enhanced panicle blast resistance. Their yield and major quality traits were synchronously improved compared to the recipient parent. Two representative lines with similar backgrounds but different regional adaptabilities have passed the variety examination and approval. This study provides a molecular design strategy for *japonica* rice improvement via a combinatorial using superior alleles of *Pigm* and *Hd1* in YRD, and the improved varieties effectively overcome the narrow regional adaptability of southern *japonica* rice.

## Figures and Tables

**Table 1:** A comparison of the main agronomic traits of the six BC_4_F_3_ lines under natural short-day conditions.

Line	Genotype	Main Agronomic Traits in 2019 Hainan							
		DTH (d)	PH (cm)	PN	PL (cm)	GNP	SSR (%)	TGW (g)	YPP (g)
ZD9471	*Hd1^H8^*	83.67 ± 0.58 a	83.60 ± 0.40 a	7.20 ± 0.17 a	14.56 ± 0.20 a	114.64 ± 5.10 b	87.68 ± 2.19 a	25.32 ± 0.23 a	23.63 ± 0.72 b
Line1	*Pigm/Hd1^H8^*	84.67 ± 0.58 a	84.20 ± 0.82 a	8.20 ± 1.32 a	14.78 ± 0.30 a	138.43 ± 7.09 a	87.35 ± 1.43 a	25.46 ± 0.38 a	26.71 ± 0.45 a
Line2	*Pigm*/*Hd1^H8^*	85.00 ± 1.00 a	83.73 ± 0.50 a	7.80 ± 0.95 a	14.45 ± 0.41 a	129.79 ± 7.04 ab	87.94 ± 1.84 a	25.45 ± 0.36 a	26.72 ± 0.89 a
Line3	*Pigm*/*Hd1^H8^*	85.67 ± 0.58 a	83.10 ± 0.95 a	8.20 ± 1.01 a	14.54 ± 0.43 a	132.32 ± 6.47 a	87.25 ± 0.54 a	25.41 ± 0.33 a	26.68 ± 0.57 a
Line4	*Pigm*/*Hd1^H13^*	85.67 ± 0.58 a	83.40 ± 0.72 a	8.00 ± 0.62 a	15.07 ± 0.27 a	129.62 ± 5.25 ab	87.55 ± 2.01 a	25.28 ± 0.33 a	26.90 ± 0.33 a
Line5	*Pigm*/*Hd1*^H13^	85.67 ± 0.58 a	83.20 ± 0.36 a	7.80 ± 0.60 a	14.86 ± 0.18 a	131.66 ± 7.35 ab	87.09 ± 0.96 a	25.26 ± 0.26 a	26.47 ± 0.82 a
Line6	*Pigm*/*Hd1^H13^*	85.00 ± 1.00 a	83.37 ± 0.70 a	7.60 ± 1.08 a	14.83 ± 0.32 a	130.98 ± 5.03 ab	87.48 ± 1.17 a	25.32 ± 0.49 a	26.76 ± 0.28 a

DTH, days to heading; PH, plant height; PN, panicles number per plant; PL, panicle length; GNP, grain number per panicle; SSR, seed setting rate; TGW, 1000-grain weight; YPP, yield per plant. Data are shown as mean ± standard deviation. Different letters in the same column indicate significant differences (*p* < 0.05). The same as below tables.

**Table 2:** A comparison of the main agronomic traits and yield of the six BC_4_F_4_ lines under natural long-day conditions.

	Genotype	Main Agronomic Traits in 2020 Jiangsu							
		DTH (d)	PH (cm)	PN	PL (cm)	GNP	SSR (%)	TGW (g)	YPP (g)
ZD9471	*Hd1^H8^*	85.67 ± 0.58 b	83.60 ± 0.40 b	8.00 ± 0.95 a	16.72 ± 0.20 b	147.02 ± 6.51 b	91.18 ± 1.64 a	24.38 ± 0.27 a	28.22 ± 0.40 c
Line1	*Pigm/Hd1^H8^*	85.67 ± 0.58 b	83.07 ± 0.25 b	8.27 ± 0.95 a	17.06 ± 0.20 b	165.24 ± 6.21 b	91.65 ± 1.51 a	24.45 ± 0.21 a	33.04 ± 2.31 c
Line2	*Pigm*/*Hd1^H8^*	86.00 ± 1.00 b	83.33 ± 0.42 b	8.27 ± 1.36 a	16.92 ± 0.10 b	159.77 ± 5.11 b	91.20 ± 1.22 a	24.51 ± 0.17 a	32.42 ± 2.42 c
Line3	*Pigm*/*Hd1^H8^*	86.67 ± 0.58 b	83.07 ± 0.55 b	7.93 ± 0.96 a	17.06 ± 0.41 b	164.93 ± 6.73 b	91.23 ± 0.31 a	24.52 ± 0.20 a	34.82 ± 4.66 bc
Line4	*Pigm*/*Hd1^H13^*	95.33 ± 0.58 a	95.20 ± 0.36 a	8.00 ± 0.85 a	17.90 ± 0.12 a	194.74 ± 5.19 a	91.88 ± 1.09 a	24.18 ± 0.08 a	43.77 ± 4.88 a
Line5	*Pigm*/*Hd1^H13^*	95.67 ± 0.58 a	95.27 ± 0.64 a	8.07 ± 1.03 a	17.91 ± 0.32 a	198.73 ± 7.75 a	91.93 ± 1.63 a	24.18 ± 0.13 a	43.87 ± 1.59 a
Line6	*Pigm*/*Hd1^H13^*	96.33 ± 0.58 a	94.77 ± 0.35 a	7.83 ± 0.84 a	17.88 ± 0.22 a	198.63 ± 8.39 a	91.41 ± 1.46 a	24.21 ± 0.30 a	42.60 ± 2.53 ab

**Table 3:** A comparison of the main quality characteristics of the six BC_4_F_4_ lines under natural long-day conditions.

Line	Genotype	Main Quality Characteristics in 2020 Jiangsu								
		BRR (%)	HRR (%)	CGP (%)	CD (%)	AC (%)	PC (%)	AS	TS	CTV
ZD9471	*Hd1^H8^*	81.95 ± 0.28 ab	69.39 ± 0.22 a	18.42 ± 0.42 a	5.23 ± 0.15 a	14.92 ± 0.12 a	10.50 ± 0.30 a	7.73 ± 0.06 b	8.13 ± 0.06 b	73.87 ± 0.06 b
Line1	*Pigm/Hd1^H8^*	81.97 ± 0.40 ab	69.15 ± 0.39 ab	16.25 ± 0.16 b	4.61 ± 0.32 b	14.84 ± 0.15 a	10.35 ± 0.17 a	7.67 ± 0.06 b	8.20 ± 0.06 b	74.07 ± 0.06 b
Line2	*Pigm/Hd1^H8^*	82.02 ± 0.41 a	69.20 ± 0.40 ab	16.43 ± 0.18 b	4.72 ± 0.28 ab	14.77 ± 0.08 a	10.31 ± 0.19 a	7.67 ± 0.06 b	8.17 ± 0.06 b	74.00 ± 0.10 b
Line3	*Pigm/Hd1^H8^*	81.94 ± 0.53 ab	69.16 ± 0.91 ab	16.44 ± 0.13 b	4.69 ± 0.25 ab	14.80 ± 0.11 a	10.23 ± 0.13 a	7.67 ± 0.06 b	8.20 ± 0.00 b	74.03 ± 0.12 b
Line4	*Pigm/Hd1^H13^*	80.93 ± 0.46 b	67.46 ± 0.26 c	7.75 ± 0.26 c	2.23 ± 0.13 c	13.96 ± 0.06 b	8.94 ± 0.05 b	9.33 ± 0.06 a	9.47 ± 0.06 a	80.40 ± 0.20 a
Line5	*Pigm/Hd1^H13^*	80.90 ± 0.25 b	67.73 ± 0.36 c	7.12 ± 0.32 cd	1.98 ± 0.08 c	13.95 ± 0.09 b	8.92 ± 0.05 b	9.37 ± 0.06 a	9.43 ± 0.06 a	80.40 ± 0.10 a
Line6	*Pigm/Hd1^H13^*	81.01 ± 0.29 ab	67.97 ± 0.47 bc	6.59 ± 0.34 d	1.75 ± 0.20 c	13.95 ± 0.06 b	8.90 ± 0.06 b	9.37 ± 0.06 a	9.43 ± 0.06 a	80.47 ± 0.12 a

BRR, brown rice rate; HRR, head rice rate; CGP, chalky grain percentage; CD, chalkiness degree; AC, amylose content; PC, protein content; AS, appearance score; TS, taste score; CTV, comprehensive taste value.

**Table 4:** Multi-location test results of the two representative new lines compared to the recipient parent ZD9471.

Traits	ZD9471	Zhendao 9042		Zhendao 9049	
		2021	2022	2021	2022
Growth period (d)	151.6	148.5	150.1	155.9	157.1
Plant height (cm)	93.5	94.6	90.4	98.1	92.6
Panicles number (M/ha)	2.9	3.2	3.0	2.6	2.9
Grain number per panicle	127.4	143.4	150.9	196.8	155.8
Seed setting rate (%)	88.6	87.2	88.5	92.3	94.5
1000-grain weight (g)	25.3	26.0	25.9	25.6	25.8
Yield (T/ha)	9.9	10.1	9.7	11.0	10.5
Brown rice rate (%)	82.2	83.8	83.3	84.4	83.0
Head rice rate (%)	71.1	74.2	73.3	75.2	72.9
Chalky grain rate (%)	17	8	3	17	3
Chalkiness degree (%)	2.4	0.5	0.3	2.3	0.3
Amylose content (%)	15.7	16.0	17.8	14.2	18.3
Gel consistency (mm)	75	84	75	70	76
Alkali spreading value	6	7	7	7	7
Quality grade	2	1	1	2	2
Leaf blast resistant index	0	0	0	0	0
Panicle blast resistant index	5	3	3	1	1
Resistance composite index	4.30	4.75	3.25	1.75	2.00
Grading incidence	MS	MS	MR	R	R

The data were derived from the results of regional trials https://www.ricedata.cn/variety/index.htm (accessed on 1 March 2025). Values represent means.

**Table 5:** Detecting markers for *Pigm* and *Hd1* employed in this study.

Marker	Forward Primer (5′-3′)	Reverse Primer (5′-3′)	Physical Location (bp)
Pigm-2	TCTGAATTATTGTGGTCGTG	CCGTTCACATCAGTTTTTCT	10,366,681–10,366,821
Pigm-4	ATGCTCGATTCGTTACATTT	CGTCCCACACTTTCTTTTT	10,436,075–10,436,229
Hd1-SNP	FAM-F1: GAAGGTGACAAGTTCATGCTGGCGAGCGGGTTCGCGGAGTA	TGCTCGCGCACGCCCACACGCTCCGG	9,336,752–9,336,854
	HEX-F2: GAAGGTCGGAGTCAACGGATTGGCGAGCGGGTTCGCGGAGTG		

## Data Availability

Data are contained within the article.

## Supplementary Materials

The following supporting information can be downloaded at https://www.mdpi.com/article/10.3390/plants14121836/s1.

## References

[1] Hua J., Zhou N., Zhang H., Huo Z., Xu K., Wei H., Gao H., Guo B., Dai Q., Zhou P. (2014). Situation and strategies of *Japonica* rice production and development in southern China. China Rice.

[2] Zhu Y., Xu D., Ma Z., Chen X., Zhang M., Zhang C., Liu G., Wei H., Zhang H. (2021). Differences in eating quality attributes between *japonica* rice from the northeast region and semi-glutinous *japonica* rice from the Yangtze river delta of China. Foods.

[3] Yan Y., Wang K., Zhou F., Zhang L., Hu Z., Cao L., Wu S. (2022). Identification and resistance evaluation of rice blast resistance genes in *japonica* rice germplasm in Yangtze river delta. J. Nucl. Agri. Sci..

[4] Qi Z., Du Y., Yu J., Zhang R., Yu M., Cao H., Song T., Pan X., Liang D., Liu Y. (2023). Molecular detection and analysis of blast resistance genes in rice main varieties in Jiangsu province, China. Agronomy.

[5] Xu M. (2020). Application status, problems and development strategies of high quality rice varieties in Jiangsu Province. Chin. Rice.

[6] Qi Z., Yu J., Zhang R., Yu M., Du Y., Cao H., Song T., Pan X., Liang D., Liu Y. (2022). Identification and evaluation of resistance of new rice cultivars (lines) and main cultivars to rice blast in Jiangsu province from 2016 to 2020. Jiangsu Agric. Sci..

[7] Xiao N., Wu Y., Li A. (2020). Strategy for use of rice blast resistance genes in rice molecular breeding. Rice Sci..

[8] Li W., Chern M., Yin J., Wang J., Chen X. (2019). Recent advances in broad-spectrum resistance to the rice blast disease. Curr. Opin. Plant. Biol..

[9] Deng Y., Zhai K., Xie Z., Yang D., Zhu X., Liu J., Wang X., Qin P., Yang Y., Zhang G. (2017). Epigenetic regulation of antagonistic receptors confers rice blast resistance with yield balance. Science.

[10] Jin Y., He N., Cheng Z., Lin S., Huang F., Wang W., Li Q., Yang D. (2025). Resistance spectrum analysis and breeding utilization of rice blast resistance gene *Pigm-1*. Plants.

[11] Yu M., Dai Z., Pan C., Chen X., Yu L., Zhang X., Li Y., Xiao N., Gong H., Sheng S. (2013). Resistance spectrum difference between two broad-spectrum blast resistance genes, *Pigm* and *Pi2*, and their interaction effect on *Pi1*. Aata Agron. Sin..

[12] Wu Y., Xiao N., Yu L., Pan C., Li Y., Zhang X., Liu G., Dai Z., Pan X., Li A. (2015). Combination patterns of major *R* genes determine the level of resistance to the *M. oryzae* in rice (*Oryza sativa* L.). PLoS ONE.

[13] Wu Y., Xiao N., Chen Y., Yu L., Pan C., Li Y., Zhang X., Huang N., Ji H., Dai Z. (2019). Comprehensive evaluation of resistance effects of pyramiding lines with different broad-spectrum resistance genes against *Magnaporthe oryzae* in rice (*Oryza sativa* L.). Rice.

[14] Feng Z., Li M., Xu Z., Gao P., Wu Y., Wu K., Zhao J., Wang X., Wang J., Li M. (2022). Development of rice variety with durable and broad-spectrum resistance to blast disease through marker-assisted introduction of *Pigm* gene. Front. Plant Sci..

[15] Gao P., Li M., Wang X., Xu Z., Wu K., Sun Q., Du H., Younas M., Zhang Y., Feng Z. (2023). Identification of elite *R*-gene combinations against blast disease in *japonica* rice varieties. Int. J. Mol. Sci..

[16] Sun C., Chen D., Fang J., Wang P., Deng X., Chu C. (2014). Understanding the genetic and epigenetic architecture in complex network of rice flowering pathways. Protein Cell.

[17] Zhou S., Zhu S., Cui S., Hou H., Wu H., Hao B., Cai L., Xu Z., Liu L., Jiang L. (2021). Transcriptional and post-transcriptional regulation of heading date in rice. New Phytol..

[18] Zhang J., Zhou X.C., Yan W.H., Zhang Z.Y., Lu L., Han Z.M., Zhao H., Liu H.Y., Song P., Hu Y. (2015). Combinations of the *Ghd7*, *Ghd8* and *Hd1* genes largely define the ecogeographical adaptation and yield potential of cultivated rice. New Phytol..

[19] Zhang Z.H., Zhu Y.J., Wang S.L., Fan Y.Y., Zhuang J.Y. (2021). Genetic interaction of *Hd1* with *Ghd7*, *DTH8* and *Hd2* largely determines Eco-geographical adaption of rice varieties in Southern China. Rice Sci..

[20] Leng Y., Gao Y., Chen L., Yang Y., Huang L., Dai L., Ren D., Xu Q., Zhang Y., Ponce K. (2020). *Heading date 1* preponderant alleles from *indica* cultivars to breed high-yield, high-quality *japonica* rice varieties for cultivation in south China. Plant Biotechnol. J..

[21] Wei X., Jiang L., Xu J., Liu X., Liu S., Zhai H., Wan J. (2009). The distribution of *japonica* rice cultivars in the lower region of the Yangtze river valley is determined by its photoperiod-sensitivity and heading date genotypes. J. Integr. Plant Biol..

[22] Dong X., Zhu Y., Chen Z., Zhang H. (2020). Yield characteristics of *japonica*/*indica* hybrids rice in the middle and lower reaches of the Yangtze river in China. J. Integr. Agric..

[23] Xiao N., Pan C., Li Y., Wu Y., Cai Y., Lu Y., Wang R., Yu L., Shi W., Kang H. (2021). Genomic insight into balancing high yield, good quality, and blast resistance of *japonica* rice. Genome Biol..

[24] Qian Q., Guo L., Smith S., Li J. (2016). Breeding high-yield superior quality hybrid super rice by rational design. Natl. Sci. Rev..

[25] Ning Y., Liu W., Wang G. (2017). Balancing immunity and yield in crop plants. Trends Plant Sci..

[26] Yano M., Katayose Y., Ashikari M., Yamanouchi U., Monna L., Fuse T., Baba T., Yamamoto K., Umehara Y., Nagamura Y. (2000). *Hd1*, a major photoperiod sensitivity quantitative trait locus in rice, is closely related to the Arabidopsis flowering time gene *CONSTANS*. Plant Cell.

[27] Liu X.Y., Zhou C., Zhao Y., Zhou S.L., Wang W.T., Zhou D.X. (2014). The rice enhancer of zeste [E(z)] genes *SDG711* and *SDG718* are respectively involved in long day and short day signaling to mediate the accurate photoperiod control of flowering time. Front. Plant Sci..

[28] Peng P., Jiang H., Luo L., Ye C., Xiao Y. (2023). Pyramiding of multiple genes to improve rice blast resistance of photo-thermo sensitive male sterile line, without yield penalty in hybrid rice production. Plants.

[29] Xie Z.E., Yang L.Y., Zhang Z.G. (2025). One stone for multiple birds: *PigmR* integrates multiple defense pathways for high and broad-spectrum blast resistance in rice. Stress Biol..

[30] Wu Y.Y., Chen Y., Pan C.H., Xiao N., Yu L., Li Y.H., Zhang X.X., Pan X.B.A., Chen X.J., Liang C.Z. (2017). Development and evaluation of near-isogenic lines with different blast resistance alleles at the *Piz* locus in *japonica* rice from the lower region of the Yangtze River, China. Plant Dis..

[31] Wang K., Fu C.J., Fu X.X., Qin P., Hu X.C., Zhang X.W., Deng Z., Yan T.Z., Jiang N., Li Y.F. (2025). Enhancing the blast resistance of an elite thermo-sensitive genic male sterile line (TGMS) Longke638S and its derived hybrid varieties by incorporating *Pigm* gene. Mol. Breed..

[32] Monna L., Lin H., Kojima S., Sasaki T., Yano M. (2002). Genetic dissection of a genomic region for a quantitative trait locus, *Hd3*, into two loci, *Hd3a* and *Hd3b*, controlling heading date in rice. Theor. Appl. Genet..

[33] Matsubara K., Ogiso-Tanaka E., Hori K., Ebana K., Ando T., Yano M. (2012). Natural variation in *Hd17*, a homolog of *Arabidopsis ELF3* that is involved in rice photoperiodic flowering. Plant. Cell. Physiol..

[34] Tian Z., Qian Q., Liu Q., Yan M., Liu X., Yan C., Liu G., Gao Z., Tang S., Zeng D. (2009). Allelic diversities in rice starch biosynthesis lead to a diverse array of rice eating and cooking qualities. Proc. Natl Acad. Sci. USA.

[35] Gao Z., Zeng D., Cheng F., Tian Z., Guo L., Su Y., Yan M., Jiang H., Dong G., Huang Y. (2011). *ALK*, the key gene for gelatinization temperature, is a modifier gene for gel consistency in rice. J. Integr. Plant Biol..

[36] Li J., Zhao X., Cheng K., Du H., Ouyang Y., Chen J., Qiu S., Huang J., Jiang Y., Jiang L. (2012). A killer-protector system regulates both hybrid sterility and segregation distortion in rice. Science.

[37] (2014). Ministry of Agriculture of the People’s Republic of China.

[38] Lu J., Jiang Z., Chen J., Xie M., Huang W., Li J., Zhuang C., Liu Z., Zheng S. (2023). SET DOMAIN GROUP 711-mediated H3K27me3 methylation of cytokinin metabolism genes regulates organ size in rice. Plant Physiol..

[39] Zeng S., Li C., Du C., Sun L., Jing D., Lin T., Yu B., Qian H., Yao W., Zhou Y. (2018). Development of specific markers for *Pigm* in marker-assisted breeding of panicle blast resistant *japonica* rice. Chin. J. Rice Sci..

[40] (2007). Examining Rules for Identification Method and Evaluating Standard of Resistance of Rice Cultivars (Lines) to Rice Blast.

[41] (2014). Ministry of Agriculture of the People’s Republic of China.

[42] (2014). Ministry of Agriculture of the People’s Republic of China.

[43] Yang Y., Guo M., Sun S., Zou Y., Yin S., Liu Y., Tang S., Gu M., Yang Z., Yan C. (2019). Natural variation of *OsGluA2* is involved in grain protein content regulation in rice. Nat. Commun..

