# Supplementary material for: The Molecular Breeding of Different Ecotype Japonica Varieties Resistant to Rice Blast with High Genome Collinearity
Source: Plants (Basel). 2025 Jun 15;14(12):1836. doi: 10.3390/plants14121836 (PMC12197099; doi:10.3390/plants14121836)
Supplement: Supplementary file 1 [file plants-14-01836-s001.zip › plants-3550304-supplementary.pdf]

Table A1. The main functional gene of the recipient parent and six introgression lines.

Chr.	Position_RGAP _ 7.0 (bp)	Gene	MSU_ID	Alt-Allele-Function	ZD9471	Line1	Line2	Line3 (ZD9042)	Line4	Line5	Line6 (ZD9049)
1	11462725	*Os-HKT1;5/SKC1*	LOC_Os01g20160	increasing salt tolerance							
1	36346000	*OsPP15*	LOC_Os01g62760	increasing drought tolerance	√	√	√	√	√	√	√
2	1663821	*NRAT1*	LOC_Os02g03900	decreasing Al tolerance							
2	1658896	*NRAT1*	LOC_Os02g03900	decreasing Al tolerance							
2	1659849	*NRAT1*	LOC_Os02g03900	decreasing Al tolerance							
2	1661026	*NRAT1*	LOC_Os02g03900	decreasing Al tolerance							
2	5410205	*OsHMA4*	LOC_Os02g10290	Grain lower Cu							
3	220113	*qLTG3-1*	LOC_Os03g01320	Low germinability at low-temperature (loss-of-function)							
4	23886659	*BET1*	LOC_Os04g40140	increasing boron-toxicity tolerance	√	√	√	√	√	√	√
4	30726195	*OsHKT1;1*	LOC_Os04g51820	increasing root sodium content							
4	33304910	*OsJAZ1*	LOC_Os04g55920	decreasing root length and weight							
6	29539670	*OsHKT2;1*	LOC_Os06g48810	lower potassium use efficiency							
7	7407917	*OsHMA3*	LOC_Os07g12900	high cadmium accumulation							
7	7409315	*OsHMA3*	LOC_Os07g12900	high cadmium accumulation							
7	8878858	*qGMN7.1/OsNRAMP5*	LOC_Os07g15370	high cadmium accumulation							
7	8879641	*qGMN7.1/OsNRAMP5*	LOC_Os07g15370	high cadmium accumulation							
7	22930113	*OsSAP16*	LOC_Os07g38240	increasing resistance to tungro spherical virus	√	√	√	√	√	√	√
8	86089	*Os-MOT1;1*	LOC_Os08g01120	decreasing molybdenum accumulation							
9	16308325	*DRO1*	LOC_Os09g26840	shallow rooting							

Chr.	Position_RGAP _ 7.0 (bp)	Gene	MSU_ID	Alt-Allele-Function	ZD9471	Line1	Line2	Line3 (ZD9042)	Line4	Line5	Line6 (ZD9049)
9	18122850	*bZIP73*	LOC_Os09g29820	decreasing chilling tolerance							
10	4666493	*qUVR-10*	LOC_Os10g08580	High CPD photolyase activity	√	√	√	√	√	√	√
10	18975900	*DROT1*	LOC_Os10g35460	increasing drought tolerance							
11	16984309	*HAN1*	LOC_Os11g29290	decreasing chilling tolerance							
12	14233796	*Nced*	LOC_Os12g24800	drought resistance							
3	18437564	*bsr-d1*	LOC_Os03g32230	increasing blast resistance							
4	6942614	*Bph3*	LOC_Os04g12540	increasing brown planthopper resistance							
4	6956655	*Bph3*	LOC_Os04g12560	increasing brown planthopper resistance							
4	6968474	*Bph3*	LOC_Os04g12580	increasing brown planthopper resistance							
4	31600315	*PiPR1*	LOC_Os04g53050	decreasing blast tolerance							
4	31602115	*PiPR1*	LOC_Os04g53050	decreasing blast tolerance							
4	31602847	*PiPR1*	LOC_Os04g53050	decreasing blast tolerance							
4	31662295	*Xa1*	LOC_Os04g53160	increasing blight resistance							
6	10389573	*Pi50*	LOC_Os06g17900	increasing blast resistance		H	H	H	H	H	H
9	9672381	*Pii/HIT7/pi5-1*	LOC_Os09g15840	increasing blast resistance							
9	9779736	*Pi56*	LOC_Os09g16000	increasing blast resistance							
11	6544171	*Pi-CO39/RGA4*	LOC_Os11g11790	increasing blast resistance							
11	7659694	*LHCB5*	LOC_Os11g13890	increasing blast resistance							
11	28399471	*Xa26/Xa3*	LOC_Os11g47210	increasing blight resistance							
11	28399720	*Xa26/Xa3*	LOC_Os11g47210	increasing blight resistance	√	√	√	√	√	√	√
12	10833368	*Ptr*	LOC_Os12g18729	increasing blast resistance							
1	36355847	*HESO1/OsHESO1*	LOC_Os01g62780	later days to headings	√	√	√	√	√	√	√
1	40363925	*OsMADS51*	LOC_Os01g69850	delaying heading date							
2	23990288	*OsCOL4*	LOC_Os02g39710	promoting heading date	N	N	N	N	N	N	N

Chr.	Position_RGAP _ 7.0 (bp)	Gene	MSU_ID	Alt-Allele-Function	ZD9471	Line1	Line2	Line3 (ZD9042)	Line4	Line5	Line6 (ZD9049)
2	23991055	*OsCOL4*	LOC_Os02g39710	promoting heading date							
2	30096330	*DTH2/Hd7*	LOC_Os02g49230	delaying heading date under LD	H	H	H	H	H	H	H
3	1270327	*OsMADS50/Hd9/OsSOC1/DTH3*	LOC_Os03g03070	delaying heading date							
3	33002789	*Hd16*	LOC_Os03g57940	promoting heading date under LD							
6	2235191	*Hd17/Hd3b*	LOC_Os06g05060	delaying heading date	√	√	√	√	√	√	√
6	2928178	*RFT1*	LOC_Os06g06300	delaying heading date							
6	2942293	*Hd3a*	LOC_Os06g06320	Late heading							
6	9337101	*Hd1*	LOC_Os06g16370	promoting heading date under LD					√	√	√
6	9337217	*Hd1*	LOC_Os06g16370	promoting heading date under LD							
6	9337239	*Hd1*	LOC_Os06g16370	promoting heading date under LD							
6	9338220	*Hd1*	LOC_Os06g16370	promoting heading date under LD							
6	9338243	*Hd1*	LOC_Os06g16370	promoting heading date under LD							
7	9152558	*Ghd7/Hd4*	LOC_Os07g15770	promoting heading date under LD							
7	29627357	*OsPRR37/Hd2/Ghd7.1*	LOC_Os07g49460	promoting heading date under LD							
7	29628481	*OsPRR37/Hd2/Ghd7.1*	LOC_Os07g49460	promoting heading date under LD							
7	29628484	*OsPRR37/Hd2/Ghd7.1*	LOC_Os07g49460	promoting heading date under LD							
7	29628529	*OsPRR37/Hd2/Ghd7.1*	LOC_Os07g49460	promoting heading date under LD							
7	29623803	*OsPRR37/Hd2/Ghd7.1*	LOC_Os07g49460	early flowering							
7	29628500	*OsPRR37/Hd2/Ghd7.1*	LOC_Os07g49460	early flowering							

Chr.	Position_RGAP _ 7.0 (bp)	Gene	MSU_ID	Alt-Allele-Function	ZD9471	Line1	Line2	Line3 (ZD9042)	Line4	Line5	Line6 (ZD9049)
8	4332836	*Ghd8*	LOC_Os08g07740	promoting heading date under LD							
8	4333871	*DTH8*	LOC_Os08g07740	promoting heading date under LD							
10	17076177	*Ehd1*	LOC_Os10g32600	delaying heading date							
11	2454121	*RCN1*	LOC_Os11g05470	delaying heading date							
11	4432786	*GATA28*	LOC_Os11g08410	later heading date,greater plant height and larger panicle length							
1	178766	*OsNPF6.1*	LOC_Os01g01360	higher nitrogen use efficiency							
1	14452823	*psr1*	LOC_Os01g25484	increasing regeneration ablility	N	H	N	H	N	H	N
1	33453244	*OsPME1*	LOC_Os01g57854	more MeOH-Jasmonates							
2	31768358	*OsTSD2*	LOC_Os02g51860	more MeOH-Jasmonates							
3	6894836	*BOC1*	LOC_Os03g12820	callus browning							
3	21067004	*qSE3/OsHAK21*	LOC_Os03g37930	increasing salt tolerance							
7	23797130	*LOC_Os07g39700*	LOC_Os07g39700	increasing sprouting							
10	21759092	*NRT1.1B*	LOC_Os10g40600	higher nitrogen use efficiency							
1	5244076	*D2/CYP90D2/SMG1 1*	LOC_Os01g10040	larger tiller angle							
1	29929465	*D61/OsBRI1*	LOC_Os01g52050	larger flag leaf angle							
1	29930698	*D61/OsBRI1*	LOC_Os01g52050	larger flag leaf angle							
1	38383141	*sd1*	LOC_Os01g66100	semi-dwarf							
3	3845747	*ILI3/OsbHLH153*	LOC_Os03g07540	increasing flag leaf angle							
3	28428731	*SCM3/OsTB1*	LOC_Os03g49880	increasing lodging resistance							
3	28513552	*SLR1/OsGAI/Slr1-d*	LOC_Os03g49990	higher plant height							
3	29586015	*TAC3*	LOC_Os03g51660	larger tiller angle							
5	6658027	*OsGSK2*	LOC_Os05g11730	increasing mesocotyl length							

Chr.	Position_RGAP _ 7.0 (bp)	Gene	MSU_ID	Alt-Allele-Function	ZD9471	Line1	Line2	Line3 (ZD9042)	Line4	Line5	Line6 (ZD9049)
6	27484286	*APO1*	LOC_Os06g45460	increasing grain number							
8	20930988	*TIG1*	LOC_Os08g33530	smaller tiller angle							
8	26887943	*OsOTUB1/WTG1*	LOC_Os08g42540	decreasing tiller number and increasing grain number							
8	27998573	*OsSPY*	LOC_Os08g44510	increasing plant height							
9	14568100	*OsTb2*	LOC_Os09g24480	increasing tiller number							
9	17184182	*LP1*	LOC_Os09g28300	long panicle							
9	20731844	*TAC1*	LOC_Os09g35980	Spread-out plant architecture							
10	13723265	*OsbHLH174*	LOC_Os10g26410	increasing flag leaf angle							
1	30712558	*OsUGT706D1*	LOC_Os01g53460	more 7-O-glucoside							
2	35370598	*Trigonelline_gwas*	LOC_Os02g57760	fewer trigonelline	√	√	√	√	√	√	√
4	6566248	*LOC_Os04g11970*	LOC_Os04g11970	more O-methylapigenin C-pentoside							
7	2188150	*LOC_Os07g04970*	LOC_Os07g04970	fewer feruloylserotonin							
7	2188914	*LOC_Os07g04970*	LOC_Os07g04970	fewer feruloylserotonin							
7	19060398	*OsUGT707A2*	LOC_Os07g32060	more 5-O-glucoside							
7	19061609	*OsUGT707A2*	LOC_Os07g32060	more 5-O-glucoside	N	N	N	N	N	N	N
9	21464008	*OsAT4c*	LOC_Os09g37200	fewer N-feruloylputrescine							
12	15999119	*LOC_Os12g27254*	LOC_Os12g27254	fewer N',N''-p-coumaroyl feruloyl spermidine							
1	25382824	*Rd/DFR/OsDfr*	LOC_Os01g44260	light red seed coat							
1	25383093	*Rd/DFR/OsDfr*	LOC_Os01g44260	red seed coat							
1	25383832	*Rd/DFR/OsDfr*	LOC_Os01g44260	light red seed coat							
1	36461792	*qSH1*	LOC_Os01g62920	seed shattering	√	√	√	√	√	√	√
2	8115620	*GW2*	LOC_Os02g14720	larger grain width and weight							

Chr.	Position_RGAP _ 7.0 (bp)	Gene	MSU_ID	Alt-Allele-Function	ZD9471	Line1	Line2	Line3 (ZD9042)	Line4	Line5	Line6 (ZD9049)
3	16733441	*GS3*		Large grain (loss-of-function)							
4	25959585	*An-2/OsLOGL6/LABA1*	LOC_Os04g43840	shorter awn							
4	25960486	*An-2/OsLOGL6/LABA1*	LOC_Os04g43840	awn present							
4	28894753	*OsCYP704A3*	LOC_Os04g48460	Longer seed size							
6	5316554	*OsC1*	LOC_Os06g10350	colorless apiculus							
6	27024794	*Hairy Leaf 6/HL6*	LOC_Os06g44750	increasing grain weight and plant height							
7	6068017	*Rc*	LOC_Os07g11020	red seed coat							
7	6068071	*Rc*	LOC_Os07g11020	red seed coat	√	√	√	√	√	√	√
1	38137703	*OsAAP6*	LOC_Os01g65670	increasing seed protein content	√	√	√	√	√	√	√
6	1633040	*OsACS6/SSG6*	LOC_Os06g03990	more L-asparagine							
6	6752756	*ALK*	LOC_Os06g12450	High gelatinization temperature	H	H	H	H	H	H	H
8	20382858	*Badh2*	LOC_Os08g32870	fragrance							
8	20380275	*Badh2*	LOC_Os08g32870	fragrance							
8	20379883	*Badh2*	LOC_Os08g32870	fragrance							
8	20385593	*Badh2*	LOC_Os08g32870	fragrance							
1	5270928	*Gn1a/OsCKX2*	LOC_Os01g10110	increasing grain number							
1	5271719	*Gn1a/OsCKX2*	LOC_Os01g10110	increasing grain number							
1	5275530	*Gn1a/OsCKX2*	LOC_Os01g10110	increasing grain number							
1	5275544	*Gn1a/OsCKX2*	LOC_Os01g10110	increasing grain number							

Chr.	Position_RGAP _ 7.0 (bp)	Gene	MSU_ID	Alt-Allele-Function	ZD9471	Line1	Line2	Line3 (ZD9042)	Line4	Line5	Line6 (ZD9049)
1	5568692	*Rf3/OsMADS3*	LOC_Os01g10504	fertility restoration							
1	18722895	*ESA1*	LOC_Os01g34010	hybrid incompatibility							
1	22376434	*SaF*	LOC_Os01g39670	non-japonica type							
1	31558876	*NOG1*	LOC_Os01g54860	increasing grain number							
1	35555900	*LAX1*	LOC_Os01g61480	increasing grain number	√	√	√	√	√	√	√
1	35558484	*LAX1*	LOC_Os01g61480	increasing grain number							
2	8117283	*GW2*	LOC_Os02g14720	larger grain width and weight							
2	9997737	*Rf2*	LOC_Os02g17380	fertility restoration							
2	28865723	*qNGR2/GRF4*	LOC_Os02g47280	larger grain size and higher nitrogen use efficiency							
3	4353347	*OsLG3*	LOC_Os03g08470	increasing drought tolerance							
3	4352797	*OsLG3*	LOC_Os03g08470	increasing drought tolerance							
3	4352960	*OsLG3*	LOC_Os03g08470	increasing drought tolerance							
3	4353103	*OsLG3*	LOC_Os03g08470	increasing drought tolerance							
3	6053303	*qLGY3/OsMADS1/GW3p6/OsLG3b*	LOC_Os03g11614	increasing grain length							
3	17340602	*GL3.2/CYP78A5*	LOC_Os03g30420	deceasing grain length							
3	28093193	*LOX-3*	LOC_Os03g49350	decreasing generation of stale flavor							
3	35390422	*GL3.3/qTGW3*	LOC_Os03g62500	increasing grain length							
3	36150781	*GNP1*	LOC_Os03g63970	increaing grain number and plant height							
4	31212801	*LSCHL4*	LOC_Os04g52479	lower grain yield							
4	31214019	*LSCHL4*	LOC_Os04g52479	lower grain yield							
5	2604473	*PTB1*	LOC_Os05g05280	increasing setting rate							

Chr.	Position_RGAP _ 7.0 (bp)	Gene	MSU_ID	Alt-Allele-Function	ZD9471	Line1	Line2	Line3 (ZD9042)	Line4	Line5	Line6 (ZD9049)
5	3444274	*GS5*	LOC_Os05g06660	decreasing grain width							
5	3444712	*GS5*	LOC_Os05g06660	decreasing grain width							
6	4201227	*DPL2*	LOC_Os06g08510	hybrid incompatibility							
6	5745402	*S5-3/ORF3*	LOC_Os06g10990	hybrid incompatibility							
6	5759744	*S5*	LOC_Os06g11010	hybrid compatibility							
6	5761369	*S5*	LOC_Os06g11010	hybrid compatibility							
6	26592357	*GW6a/OsglHAT1*	LOC_Os06g44100	increasing grain weight and plant height							
6	5758523	*S5-4/ORF4*		hybrid incompatibility							
7	7545012	*OsSNB*	LOC_Os07g13170	increasing grain length							
7	15787003	*S7*	LOC_Os07g27180	hybrid compatibility							
7	19103249	*OsSPL13/GLW7*	LOC_Os07g32170	increasing grain size							
7	24669233	*GW7*	LOC_Os07g41200	increasing grain length							
7	24714487	*GE/CYP78A13/BG2*	LOC_Os07g41240	increasing grain size							
7	6693475	*Pms1/PMS1T*		photoperiod-sensitive male sterility							
8	26505387	*GW8/OsSPL16*	LOC_Os08g41940	increasing grain width		N		N		N	
8	26501201	*GW8/OsSPL16*	LOC_Os08g41940	increasing grain width		N		N		N	
8	26501258	*GW8/OsSPL16*	LOC_Os08g41940	increasing grain width							
12	24669797	*HSA1b*	LOC_Os12g39920	hybrid incompatibility							

"N" represents no signal, "H" represents heterozygous, "√" represents carry the Alt-Allele-Function, while blanks represent the absence of the allele.
